# Metagenomic next-generation sequencing-assisted diagnosis of a rare case of primary cutaneous acanthamoebiasis in an HIV patient: a case report

**DOI:** 10.3389/fcimb.2024.1356095

**Published:** 2024-05-28

**Authors:** Wei Fan, Pin Li, Qihao Wei, Xinru Liu, Yuxiang Cai, Bin Li, Yaping Lu

**Affiliations:** ^1^Department of Pathology, Zhongnan Hospital of Wuhan University, Wuhan, China; ^2^Research and Development Department, Sinopharm Medical Laboratory (Wuhan) Co., Ltd., Wuhan, China; ^3^Research and Development Department, Sinopharm Genomics Technology Co., Ltd., Wuhan, China

**Keywords:** *Acanthamoeba*, cutaneous acanthamoebiasis, skin ulcer, HIV, metagenomic nextgeneration sequencing

## Abstract

Pathogenic and free-living *Acanthamoeba* are widely distributed in the environment and have been reported to cause keratitis and universally fatal encephalitis. Primary cutaneous acanthamoebiasis caused by *Acanthamoeba* is exceedingly rare and presents as isolated necrotic cutaneous lesions without involvement of the cornea or central nervous system. Cutaneous acanthamoebiasis often occurs in immunocompromised patients and is likely overlooked or even misdiagnosed only by cutaneous biopsy tissue histopathological analysis. Here, we report a HIV-infected 63-year-old female with oral leukoplakia for 4 months and scattered large skin ulcers all over the body for 2 months. The cause of the cutaneous lesions was unclear through cutaneous specimens histopathological analysis, and subsequently *Acanthamoeba* were detected by metagenomic next-generation sequencing (mNGS), which may be the cause of cutaneous lesions. Based on the mNGS results, a pathologist subsequently reviewed the previous pathological slides and found trophozoites of *Acanthamoeba* so that the cause was identified, and the skin ulcers improved significantly after treatment with multi-drug combination therapy. *Acanthamoeba* is also a host of pathogenic microorganisms. The presence of endosymbionts enhances the pathogenicity of *Acanthamoeba*, and no other pathogens were reported in this case. mNGS is helpful for rapidly diagnosing the etiology of rare skin diseases and can indicate the presence or absence of commensal microorganisms.

## 1 Introduction

*Acanthamoeba* is widely present in the air and soil and can be isolated from swimming pools, water pipes, and even from the oral and nasal mucosa of healthy people (Siddiqui and Khan, 2012; Borecka et al., 2020). The life cycle of *Acanthamoeba* has two phases: cyst and trophozoite stage. Trophozoites are the active form, and cysts are the dormant form. *Acanthamoeba* species are opportunistic and nonopportunistic pathogens that often cause severe and even fatal consequences. Lens wearers are predisposed to amoebic infection, which may lead to amoebic keratitis and blindness. *Acanthamoeba* infection involving the central nervous system leads to amoebic encephalitis and brain necrosis. Its prognosis is poor, and most patients die within less than one month after the patient has experienced neurological symptoms (Marciano-Cabral and Cabral, 2003).

The pathogenic mechanism of *Acanthamoeba* infection is still unclear and may be related to the host immune status. Interestingly, immunocompromised patients present simple cutaneous lesions with a good prognosis, while immunocompetence usually leads to serious consequences (Galarza et al., 2009).

*Acanthamoeba* species are also hosts of pathogenic microorganisms. Bacteria, fungi, and viruses can live symbiotically within *Acanthamoeba* (Rayamajhee et al., 2021). Interactions between *Acanthamoeba* and endosymbionts exhibit variable capabilities, and it has been shown that the presence of endosymbionts enhances the pathogenicity of *Acanthamoeba* (Purssell et al., 2017). According to previous studies of commensal pathogens of *Acanthamoeba* keratitis, endosymbiotic bacteria may enhance corneal epithelial damage, increasing stromal infiltration and epithelial defects. These pathogens divide and survive within the cytoplasm of *Acanthamoeba*, and their interaction with the amoeba may be clinically significant (Rayamajhee et al., 2022).

As an emerging technology in recent years, mNGS can detect multiple microorganisms at one time, without preassuming pathogens and quickly locating suspected pathogenic microorganisms, and then provides evidence-based, reference information about diagnosis, drugs and toxicology for clinicians. Currently, this technique plays an important role in the diagnosis of fever of unknown origin (FUO), acute and severe symptoms, and difficult infections. Here, we report the case of a 63-year-old female patient infected with HIV who presented with oral leukoplakia and giant skin ulcers. *Acanthamoeba* was detected through mNGS, and the skin ulcers improved after treatment.

## 2 Case presentation

A 63-year-old female patient had 4-month history of oral leukoplakia with 2-month nonhealing, large cutaneous ulcerations all over the body. The patient was diagnosed with HIV infection 2 years ago. Physical examination revealed numerous cases of leukoplakia on the oral mucosa and a 1 cm fistula on the palate. The scattered skin ulcers were mainly on the bilateral buttocks and proximal extremities, and the largest ulcer was approximately 4*4 cm in the right buttock. The patient visited the local county hospital four months prior for oral leukoplakia and was considered to have an oral fungal infection. Oral leukoplakia improved after antifungal symptomatic treatment. However, the tumor recurred after drug withdrawal, and the hard palate abscess gradually ruptured. Three months later, oral leukoplakia recurred accompanied by multiple skin lumps, so she sought treatment again.

A series of laboratory tests were performed. The blood test results were as follows: white blood cell count 2.30*10^9^/L, red blood cell count 2.38*10^12^/L, and hemoglobin level 74 g/L. The absolute lymphocyte count was 0.2*10^9^/L. The absolute count of CD4 T lymphocyte was 4 cells/μl. Her C-reactive protein (CRP) level was 21.7 mg/L. The erythrocyte sedimentation rate (ESR) was 27 mm/h. Her procalcitonin level was normal. The activated partial thromboplastin time was 38 seconds, the thrombin time was 19.5 seconds, and the fibrinogen concentration was 148 mg/dL. The patient was herpes simplex virus type I and II IgG positive according to the TOUCH test. The interleukin 6 concentration was 45.2 pg/mL. Lung CT revealed pulmonary infection, pericardial effusion, left axillary lymph node enlargement, and aortic and coronary atherosclerosis. Abdominal CT showed no obvious abnormalities. Brain CT revealed small ischemic lesions in the left basal ganglia, partial tissue defects in the hard palate and nasal septum, and sinusitis. Brain MRI revealed left basal ganglia lacunae, white matter hyperintensity, and Fazekas grade 1.

## 3 Initial treatment

The first attempt was to temporarily administer sulfanilamide to prevent infection, and the second was to administer levofloxacin to prevent bacterial infection. Oral leukemia improved through treatment with sodium bicarbonate and Kangfuxin mouthwash. During treatment, the patient experienced intermittent fever, cough, and headache, and still, she had symptoms of nasal reflux; however, with diclofenac suppository treatment, her temperature returned to normal. The next step was to identify the cause of the skin lesions.

## 4 mNGS testing

**Figure 1:**
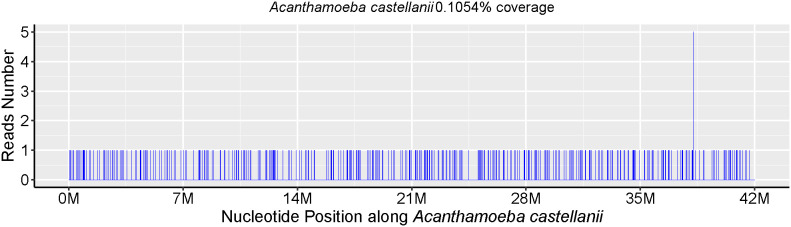
The metagenomic next-generation sequencing (mNGS) results of this patient.

Skin biopsy and mNGS were performed at the same time. Pathology revealed that part of the tissue was necrotic, chronic suppurative inflammation with abscess formation and local granulomatous inflammation with multinucleated giant cell reaction. Moreover, all the special staining results, PAS staining, Gram staining, silver staining and acid-fast staining, were negative. The parasite *Acanthamoeba* castellani, which belongs to the genus *Acanthamoeba*, was subsequently detected through mNGS without other pathogens being detected (**Table 1**), mNGS of skin tissue identified 238 sequence reads corresponding to *Acanthamoeba* castellanii (**Figure 1**).

**Table 1:** Part of the mNGS test report.

Categories	Genus			Species		
Name	Name	Reads count	Relative abundance (%)	Name	Reads count	Relative abundance (%)
Parasites	*Acanthamoeba*	412	44.98	*Acanthamoeba* castellanii	238	25.98

## 5 Follow-up and outcomes

**Figure 2:**
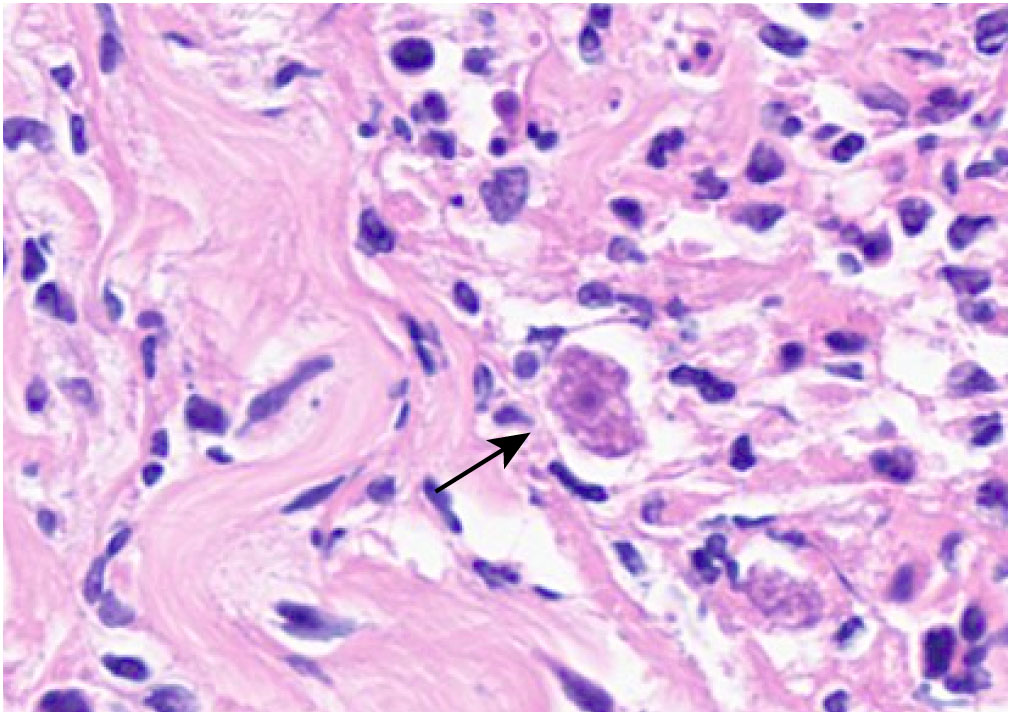
Trophozoites (black arrow) can be observed in the skin ulcer tissue (light microscope, H&E, ×400).

**Figure 3:**
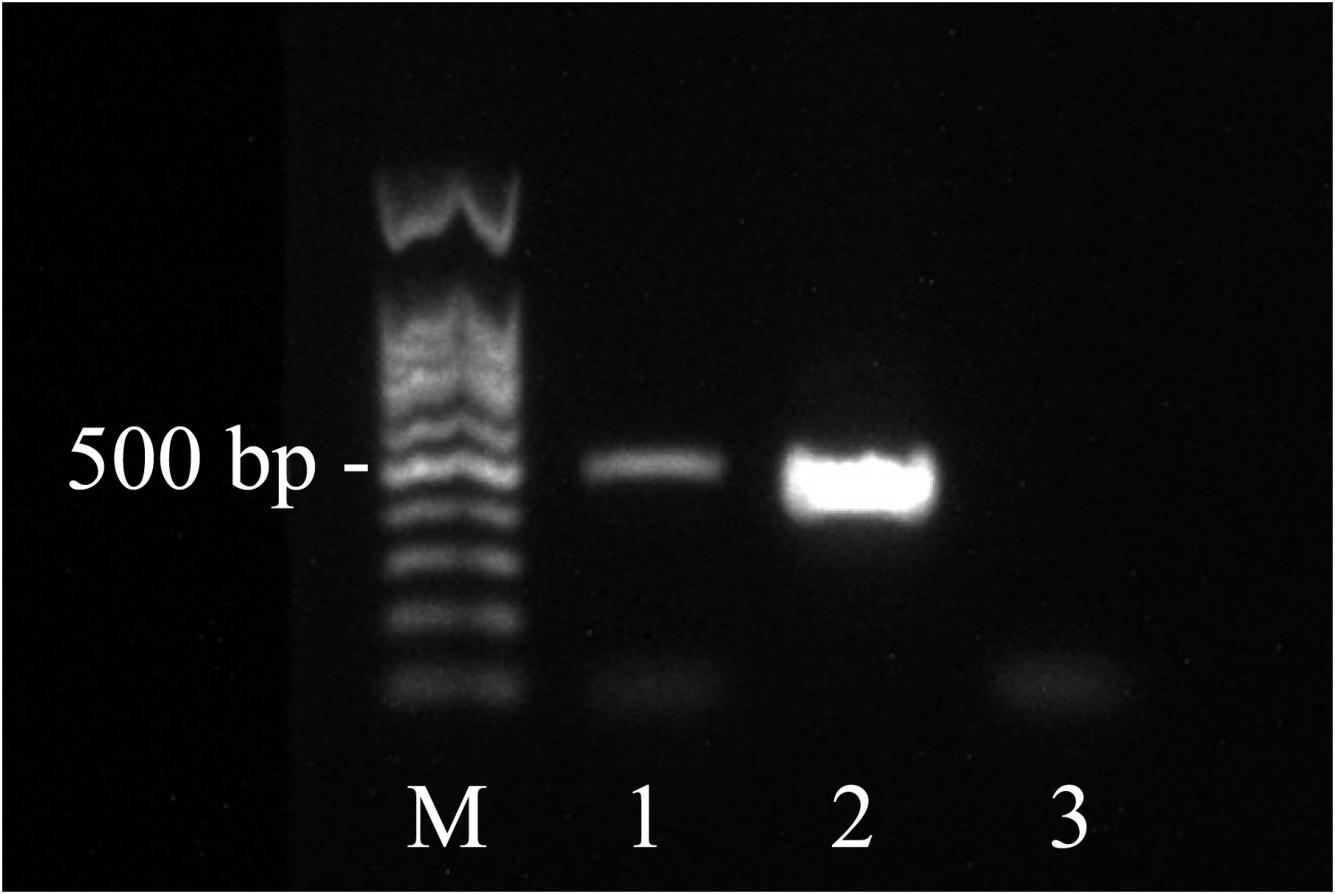
Agarose electrophoresis showing amplification of *Acanthamoeba*. (Lanes 1 =  PCR product of the test sample, Lane M = 100 bp DNA ladder, Lane 2 = positive control plasmid, Lane 3 = negative control.).

Based on the mNGS results, the pathologists subsequently reviewed the previous pathological slides and found trophozoites of *Acanthamoeba* (**Figure 2**). The laboratory used the previously reported *Acanthamoeba*-specific primers JDP1 (5′-GGCCCAGATCGTTTACCGTGAA-3′) and JDP2 (5′-TCTCAAGCTGCTAGGGGAGTCA-3′) to specifically amplify *Acanthamoeba* nucleic acid fragments (Schroeder et al., 2001). Agarose gel electrophoresis confirmed that the amplified target was consistent with the positive control plasmid (**Figure 3**). Sequence used to construct positive control plasmid was obtained from 18S rRNA gene of *Acanthamoeba* castellanii neff strain. The sequence was downloaded from GenBank database and synthesized by oligonucleotide manufacturer, the GenBank accession number was U07416.1. The amplified fragment was then subjected to Sanger sequencing, and the sequence was identified as belonging to the T4 genotype through online BLAST analysis.

Combined with the pathological and mNGS results, the clinical doctors gave sulfamethoxazole, voriconazole and metronidazole combination anti-amebic treatment. After multi-drug combination therapy, the skin ulcer dried up and crusted, and subcutaneous mass was significantly smaller than before. The patient was discharged after the afebrile and skin ulcers were better than before.

## 6 Discussion

Although *Acanthamoeba* trophozoites have a very characteristic donut-like nucleus due to their large central karyosome, it is sometimes difficult to differentiate them from macrophages solely by histopathology and they may be unrecognized or even misdiagnosed (Murakawa et al., 1995; Morrison et al., 2016). This pathogen was rapidly mapped via mNGS technology, and early detection of infectious pathogens is also conducive to early treatment. The brain MSI results showed no involvement of the central nervous system, and this patient responded well to anti-amoeba drug therapy, which was consistent with the findings of previous studies. Isolated primary cutaneous *Acanthamoeba* strains have been very rare. Cutaneous acanthamoebiasis is susceptible in immunocompromised patients, the amoebae enter through the skin or the respiratory tract, it disseminate systemically and even invade the central nervous system. Once the central nervous system is infected, therapeutic options are limited and the prognosis is grave (Singhal et al., 2001). The prognosis of cutaneous acanthamoebiasis is related to the infective dose and virulence of *Acanthamoeba*, the immune status of the host, and the timely treatment. In previous literature, the prognosis of cutaneous acanthamoebiasis is worse in immunocompetent patients than in immunocompromised ones (Sharma et al., 2017). *Acanthamoeba* species usually cause isolated primary cutaneous infection in immunocompromised patients, whereas in immunocompetent people, the initial cutaneous infection will progressed to fatal granulomatous amebic encephalitis without rapid diagnose and treatment (Paltiel et al., 2004). In the past, the diagnosis of cutaneous *Acanthamoeba* usually needed to be confirmed by amplifying the target fragment of *Acanthamoeba*-specific nucleic acid, and this scheme requires clinicians to prejudge the suspected pathogens. The mNGS-based detection scheme does not need to be preset in advance, and the method has high specificity and can quickly locate infectious pathogens, which has shown great value in the diagnosis of rare skin diseases (Stetkevich et al., 2022).

The *Acanthamoeba* genotype in this patient in the Wuhan area was T4, which is the same as that previously reported for Hong Kong; 90% of the detected *Acanthamoeba* were of the T4 genotype (Booton et al., 2002). Similar cases have not been previously reported from the Chinese mainland. Because metagenomics can detect the characteristics of bacterial, fungal, and viral parasites at the same time, mNGS is ideal for detecting symbiotic microorganisms, although only *Acanthamoeba* was detected in this case.

## Data availability statement

The raw data supporting the conclusions of this article will be made available by the authors, without undue reservation.

## Ethics statement

Written informed consent was obtained from the individual(s) for the publication of any potentially identifiable images or data included in this article. Written informed consent was obtained from the participant/patient(s) for the publication of this case report.

## Author contributions

WF: Writing – review & editing. PL: Writing – original draft. QW: Data curation, Writing – review & editing. XL: Investigation, Writing – review & editing. BL: Validation, Writing – review & editing. YC: Project administration, Writing – review & editing. YL: Writing – review & editing.
